# Local Peaks Search Method for Solving Lamb Waves’ Dispersion Equation of Laminated Structures and the Application

**DOI:** 10.3390/s23239359

**Published:** 2023-11-23

**Authors:** Jiayuan Gong, Hongyang Chen

**Affiliations:** 1 Harbin Engineering University, Harbin 150001, China; 2 Institute of Automotive Engineers, Hubei University of Automotive Technology, Shiyan 442002, China; 3 Hanjiang National Laboratory, Wuhan 430000, China; 4 Shiyan Industry Technique Academy of Chinese Academy of Engineering, Shiyan 442002, China

**Keywords:** Lamb waves, local peaks search method, dispersion equation, elastic plates, laminated structures, highlight points, sound transducers

## Abstract

To study the acoustic characteristics of sound scattered from laminated structures such as elastic plates and shells, it is usually required to solve the Lamb waves’ dispersion equations. Many traditional root-finding methods such as bisection, the Newton–Raphson method, and the Muller method are not able to tackle the problem completely. A simple but powerful method named local peaks search (LPS) is proposed to overcome their drawbacks. Firstly, the non-zero part of the dispersion equation is defined as the dispersion function, and its reciprocal is used to transform the zeros (i.e., roots) into local peaks. Secondly, the chosen complex domain is discretized, and the coarse local domains where the local peaks exist are determined by the direct search method globally. Thirdly, the Muller method is applied to obtain the refined locations of local peaks. Lastly, in order to refine the results, a hierarchical scheme is designed and the iteration of the above procedures is implemented; the error is set to be 10^−16^ as the stop criteria. The accuracy of the LPS method is validated by comparing it with the bisection method for the problem of elastic plates in the vacuum. The acoustic echo structures are analyzed experimentally. By computation of Lamb waves’ phase velocity, the critical angles are derived numerically and compared with the results acquired by an experiment using monostatic sound transducers. In this way, it is validated that the elastic scattered wave components are the highlights shown in the time-angle figure. Furthermore, the work can be applied for non-destructive testing, especially underwater structural health monitoring.

## 1. Introduction

The acoustic characteristics of sound propagated in or scattered from plates, shells, and other laminated structures have strong relationships with Lamb waves. In underwater acoustics, Lamb waves are important to study the highlights of elastic echoes scattered from structures [1,2,3,4]. Solving the dispersion equations is the foundational requirement; otherwise, the studies on acoustic characteristics are not able even to begin.

The problem of Lamb waves in a thin elastic plate with a liquid load was first studied by Osborne and Hart in 1945; the results could be expressed in terms of symmetric and anti-symmetric modes [1]. Zhu and Wu considered the viscosity of the fluid and derived the dispersion equation for Lamb waves in thin plates with viscous fluid layers [2]. Li et al. used the micro-perturbation method to approximate the load layer as appropriate boundary conditions and obtained a first-order approximate dispersion equation suitable for various thin film loads [3]. Nayfeh and Nagy improved the work of Zhu and Wu by discussing three different models for viscous fluids. They calculated the dispersion equations for leaky Lamb waves in thin plates with viscous fluid layers under several different circumstances [4]. Simonetti and Cawley studied the dispersion characteristics of SH waves in thin plates with viscoelastic layers [5,6]. Lu et al. studied the propagation characteristics of Lamb waves in a thin plate with a viscoelastic thin layer on one side and proposed a method for determining the complex Lamé constants of viscoelastic films, which involves determining *μ*_re_ by SH waves and determining *μ*_im_ by Lamb waves [7]. In these studies, there is widespread exploration of Lamb waves’ applications; however, the method for solving the Lamb wave equations has not been explicitly elucidated, posing a significant challenge in deriving dispersion and attenuation curves when commencing research in this domain.

The dispersion equation of Lamb waves is a transcendental equation that cannot be solved analytically. Root-finding methods, such as the bisection method and Newton–Raphson method, are widely implemented to obtain the real roots [8,9]. However, for many problems, the dispersion equations are complex, whose roots are also complex, which makes the problem much more difficult to solve. Barshinger and Rose proposed a minimization algorithm with real roots as the initial values to obtain the complex roots of the dispersion equation [10]. Ross applied the iterative method to solve the real roots of the dispersion equation and introduced the Muller method for solving complex roots. Muller’s method is proposed as a numerical root-finding algorithm for solving equations of the form *f*(*z*) = 0, where *z* can be real or complex. It was first presented by D. E. Muller in 1956 and is faster than the bisection method and overcomes one of the biggest drawbacks of the Newton–Raphson method, i.e., computation of derivatives at each step [11]. Liu et al. used the function ‘fminbnd’ in MATLAB to find the minimum points based on the matrix representation of the dispersion equation [12]. Some researchers used the winding number integral method to solve the complex roots of the dispersion equation. This method is based on the generalized residue theorem, which divides the complex plane into rectangular grids, determines the number of complex roots in the rectangular boundary first, and then combines algebraic theorems to obtain the complex roots of the equation [13,14]. However, the winding number integral method is rather difficult to implement and not stable. By investigation, it can be found that these methods applied for analytical formulation of the Lamb waves’ dispersion equation have their own drawbacks and are not be able to tackle with the problem completely, especially without missing any roots.

Recently, purely numerical methods such as the finite difference method, the finite element method, and the boundary element method have been studied by researchers. Uhl et al. used the finite difference method, which is a generalized semi-analytical method, to compute the dispersive curves. The method was proved stable for the considered frequency range (0~8 MHz·mm) [15]. Lamb waves within a sandwich skin were simulated based on the finite element method, and ABAQUS Software was used to extract the dispersion curve [16]. Semi-analytical formulations of Lamb waves and the dispersion curves were obtained by the scaled boundary finite element method (SBFEM), which combines advantages of the finite element and boundary element methods simultaneously [17]. Pablo Gómez et al. used COMSOL Multiphysics to generate pseudo-experimental data and then used the MASW (multichannel analysis of surface waves) method to obtain the dispersion curves of Lamb waves in plates [18]. However, these methods are difficult to implement and unstable and require a large number of elements, which makes the algorithm inefficient. Actually, these purely numerical methods are much more suitable for problems in which the analytic formulations of the dispersion equations cannot be derived. For Lamb waves in laminated structures, the analytical formulation of the dispersion equation can be expressed as a determinant. Therefore, root-finding methods are preferred for problems of Lamb waves concerning layered plates or laminated structures. However, since existing root-finding methods cannot deal with the problems perfectly, the LPS method is proposed to overcome their drawbacks while keeping their advantages.

In this paper, elastic plates are studied as the laminated structures. The local peaks search method is established, which does not require any characteristics of the dispersion function such as the derivatives, orthogonality, etc. The only requirement is that the absolute value of the complex function is continuous and can be computed accurately, which is the natural characteristic of the problem. In Section 2, the theory of Lamb waves is expressed in detail, and the dispersion equations are derived. In Section 3, the method of local peaks search is proposed, and the procedures are discussed thoroughly. In Section 4, the software is described in detail, and the algorithm of the method is further discussed. In Section 5, the LPS method is validated, and a problem of Lamb waves is computed; dispersion and attenuation curves are obtained. In Section 6, the structures of acoustic echoes scattered from an elastic plate are analyzed experimentally by monostatic sonars, and the critical angle is computed by the LPS method and compared with experimental results.

## 2. Theory

The problem of acoustic propagation in elastic bodies can be divided into two categories: bulk waves and guided waves, both of which are based on elastic theory and satisfy the same wave equation. Bulk waves are waves propagating in an infinitely elastic medium, without the constraints of boundary conditions, and can be decomposed into L-waves, SH-waves, and SV-waves. Guided waves are waves that propagate along a waveguide and are synthesized by bulk waves passing through boundary conditions. Therefore, certain boundary conditions must be met. It is the limitation of boundary conditions that makes the solution of guided wave problems very difficult. Some guided wave problems with simpler boundary conditions have been solved, such as Rayleigh waves, Lamb waves, Stoneley waves, Love waves, and Scholte waves. These guided waves are also known as surface waves or interface waves.

**Figure 1:**
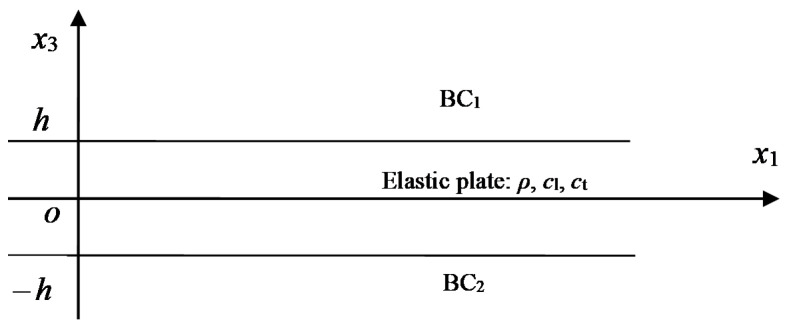
The Lamb wave model for elastic plates under diverse complicated boundary conditions.

The problem shown in Figure 1 can be used to express many diverse boundary conditions for the Lamb waves in the elastic plate. The two boundary conditions of BC1 and BC2 can be any one of air, vacuum, and water and the like fluid materials. On the elastic plate, there usually exists a viscoelastic layer on one side, which is the usage of acoustic stealth. All of these different fluids or materials will lead to a difficult problem for solving the complex dispersion equation because the roots are all complex. Especially for the problem under diverse boundary conditions, a universal method is still needed.

For guided wave propagation in elastic plates, the displacement wave equation is used, which can be written as

(1) $$\left(\lambda +\mu \right)\frac{\partial^{2}u_{j}}{\partial x_{i}\partial x_{j}}+\mu \frac{\partial^{2}u_{i}}{\partial x_{j}^{2}}+\rho f_{i}=\rho \frac{\partial^{2}u_{i}}{\partial t^{2}}$$

in which, i,j,k=1,2,3, which represents the *x*, *y*, and *z* axis. λ and μ are the Lamé constants. This equation can be used for elastic and viscoelastic materials.

In order to solve the problem, the potential function is used to derive the displacement, which has the following form:

(2) u=∇Φ+∇×Ψ

in which it can be seen that the displacement vector u can be decomposed into two components: one is the scaler potential Φ and the other is the vector potential Ψ.

For the problems shown in Figure 1, the strain is considered to be in a plane, thus the vector potential can be reduced to one dimension, that is, $\Psi =\Psi_{2}(x_{1},x_{3})j$. In this way, the problem is largely simplified. The displacement potential function can be derived as

(3) $$\left\{ \begin{array}{l} u_{1}=\frac{\partial \phi }{\partial x_{1}}-\frac{\partial \psi }{\partial x_{3}}\\ u_{2}=0\\ u_{3}=\frac{\partial \phi }{\partial x_{3}}+\frac{\partial \psi }{\partial x_{1}} \end{array}\right.$$

in which it can be seen that the second term $u_{2}$ is zero.

Based on the knowledge of the plane problem, the strain can be written as

(4) $$\left\{ \begin{array}{l} \sigma_{31}=\mu \left(\frac{\partial^{2}\phi }{\partial x_{1}\partial x_{3}}-\frac{\partial^{2}\psi }{\partial x_{3}^{2}}+\frac{\partial^{2}\psi }{\partial x_{1}^{2}}\right)\\ \sigma_{32}=0\\ \sigma_{33}=\lambda \left(\frac{\partial^{2}\phi }{\partial x_{1}^{2}}+\frac{\partial^{2}\phi }{\partial x_{3}^{2}}\right)+2\mu \left(\frac{\partial^{2}\phi }{\partial x_{3}^{2}}+\frac{\partial^{2}\psi }{\partial x_{1}\partial x_{3}}\right) \end{array}\right.$$

In general, the solution of the equation can be represented by formulation of plane waves

(5) $$\begin{array}{l} \phi =\phi \left(x_{3}\right)\exp\left[-i\left(\omega t-\xi x_{1}\right)\right]\\ \psi =\psi \left(x_{3}\right)\exp\left[-i\left(\omega t-\xi x_{1}\right)\right] \end{array}$$

in which, $\alpha^{2}=\frac{\omega^{2}}{c_{L}^{2}}-\xi^{2}$, $\beta^{2}=\frac{\omega^{2}}{c_{T}^{2}}-\xi^{2}$.

Next, the formula solution of displacement is

(6) $$\left\{ \begin{array}{l} u_{1}=i\xi A_{l}\cos(\alpha x_{3})+i\xi B_{l}\sin(\alpha x_{3})\\ +\beta A_{t}\sin(\beta x_{3})-\beta B_{t}\cos(\beta x_{3})\\ u_{3}=-\alpha A_{l}\sin(\alpha x_{3})+\alpha B_{l}\cos(\alpha x_{3})\\ +i\xi A_{t}\cos(\beta x_{3})+i\xi B_{t}\sin(\beta x_{3}) \end{array}\right.$$

and the plane wave formula solution of the strain is

(7) $$\left\{ \begin{array}{l} \sigma_{31}=\mu \{-2i\xi \alpha A_{l}\sin\left(\alpha x_{3}\right)+2i\xi \alpha B_{l}\cos\left(\alpha x_{3}\right)\\ +\left(\beta^{2}-\xi^{2}\right)A_{t}\cos\left(\beta x_{3}\right)+\left(\beta^{2}-\xi^{2}\right)B_{t}\sin\left(\beta x_{3}\right)\}\\ \sigma_{33}=\mu \{-\left(\beta^{2}-\xi^{2}\right)A_{l}\cos\left(\alpha x_{3}\right)-\left(\beta^{2}-\xi^{2}\right)B_{l}\sin\left(\alpha x_{3}\right)\\ -2i\xi \beta A_{t}\sin\left(\beta x_{3}\right)+2i\xi \beta B_{t}\cos\left(\beta x_{3}\right)\} \end{array}\right.$$

in which the time factor $\exp\left(-i\left(\omega t-\xi x_{1}\right)\right)$ is omitted.

For viscoelastic materials, the complex Young’s modulus is defined as $E^{*}=E\left(1+i\eta_{E}\right)$, *E* is the real Young’s modulus, and $\eta_{E}$ is the loss factor; the relationship with complex Lame constants $\lambda^{*}$ and $\mu^{*}$ can be derived, that is,

(8) $$\lambda^{*}=\frac{E^{*}\sigma }{\left(1+\sigma \right)\left(1-2\sigma \right)},\;\mu^{*}=\frac{E^{*}}{2\left(1+\sigma \right)}$$

The acoustic parameters of the viscoelastic media can be defined as the complex longitudinal wave speed and transverse wave speed,

(9) $$c_{L}^{*}=\sqrt{\frac{\left(\lambda^{*}+2\mu^{*}\right)}{\rho }},\;c_{T}^{*}=\sqrt{\frac{\mu^{*}}{\rho }}$$

It can also introduce the attenuation coefficients as the acoustic parameters; that is, the complex longitudinal wavenumber and the complex transverse wavenumber are defined as

(10) $$k_{L}^{*}=\frac{\omega }{c_{L}^{*}}=\frac{\omega }{c_{L}}+i\alpha_{L}$$

(11) $$k_{T}^{*}=\frac{\omega }{c_{T}^{*}}=\frac{\omega }{c_{T}}+i\alpha_{T}$$

in which $c_{L}$ is the longitudinal wave speed, $\alpha_{L}$ is the longitudinal attenuation coefficient, $c_{T}$ is the transverse wave speed, and $\alpha_{T}$ is the transverse attenuation coefficient. Obviously, $\alpha_{L}$ and $\alpha_{T}$ are the image part of the corresponding wavenumber.

For the viscoelastic layer, the formal solution of the displacement potential function can be expressed as

(12) $$\begin{array}{l} \phi^{*}=\phi^{*}\left(x_{3}\right)\exp\left[-i\left(\omega t-\xi x_{1}\right)\right]\\ \psi^{*}=\psi^{*}\left(x_{3}\right)\exp\left[-i\left(\omega t-\xi x_{1}\right)\right] \end{array}$$

Substituted it into the wave equation, we can obtain

(13) $$\begin{array}{l} \phi^{*}(x_{3})=A_{l}^{*}\cos(\alpha^{*}x_{3})+B_{l}^{*}\sin(\alpha^{*}x_{3})\\ \psi^{*}(x_{3})=A_{t}^{*}\cos(\beta^{*}x_{3})+B_{t}^{*}\sin(\beta^{*}x_{3}) \end{array}$$

in which $\alpha^{*2}=\frac{\omega^{2}}{c_{L}^{*2}}-\xi^{2},\beta^{*2}=\frac{\omega^{2}}{c_{T}^{*2}}-\xi^{2}$.

In fluid, the wave equation of displacement potential function is

(14) $$\nabla^{2}\Phi^{L}-\frac{1}{{\left(c^{L}\right)}^{2}}\frac{\partial^{2}\Phi^{L}}{\partial t^{2}}=0$$

under which u=∇Φ can be assumed. The displacement and sound pressure are written as

(15) $$\left\{ \begin{array}{l} u_{1}^{L}=\frac{\partial \Phi^{L}}{\partial x_{1}}\\ u_{3}^{L}=\frac{\partial \Phi^{L}}{\partial x_{3}}\\ p^{L}=-\rho^{L}\frac{\partial^{2}\Phi^{L}}{\partial t^{2}} \end{array}\right.$$

In fluid 1, assume the form solutions of displacement function, the displace, and the sound pressure are

(16) $$\left\{ \begin{array}{l} \Phi_{1}=A_{1}\exp(i\eta_{1}(x_{3}-h))\\ u_{3}^{1}=i\eta_{1}A_{1}\exp\left[i\eta_{1}\left(x_{3}-(h+d)\right)\right]\\ p_{1}=\rho_{1}\omega^{2}A_{1}\exp\left[i\eta_{1}\left(x_{3}-(h+d)\right)\right] \end{array}\right.$$

in which η12=ω2c12−ξ2.

In fluid 2, the formal solutions of displacement function, the displace, and the sound pressure are expressed as

(17) $$\left\{ \begin{array}{l} \phi_{2}(x_{3})=A_{2}\exp\left[-i\eta_{2}\left(x_{3}+h\right)\right]\\ u_{3}^{2}=-i\eta_{2}A_{2}\exp\left[-i\eta_{2}\left(x_{3}+h\right)\right]\\ p_{2}=\rho_{2}\omega^{2}A_{2}\exp\left[-i\eta_{2}\left(x_{3}+h\right)\right] \end{array}\right.$$

in which η22=ω2c22−ξ2.

Equations (6), (7), (12), (13), (16) and (17) are combined to derive the Lamb waves’ dispersion equation under diverse boundary conditions, especially the very complicated one. Eventually, the dispersion equation can be written as

(18) f(z)=0

where f(z) is denominated as the dispersion function.

To solve dispersion Equation (18), it is necessary to find the zeros of f(z). When the boundary condition becomes complicated, the dispersion equation will be rather difficult to solve. 

## 3. Method

Since the bisection method and Muller method cannot calculate the complex roots of the complex dispersion equations with complicated diverse boundary conditions, a novel method nominated as the local peaks search (LPS) method is proposed, which overcomes the drawbacks of Muller method while keeping its advantages. The LPS method is also a root-finding method; it searches all the local peaks and utilizes the Muller method to obtain the solutions of the dispersion equations by using a hierarchical scheme. 

The zeros of complex function *f*(*z*) can be solved by the LPS method by introducing a reciprocal function *g*(*z*):

(19) $$g\left(z\right)=\frac{1}{1+\left|f\left(z\right)\right|}$$

in which it can be seen that the zeros of *f*(*z*) are the poles of *g*(*z*). In Equation (19), the absolute value of *f*(*z*) is added by one to prevent overflow; that is, make sure the denominator is not zero.

**Figure 2:**
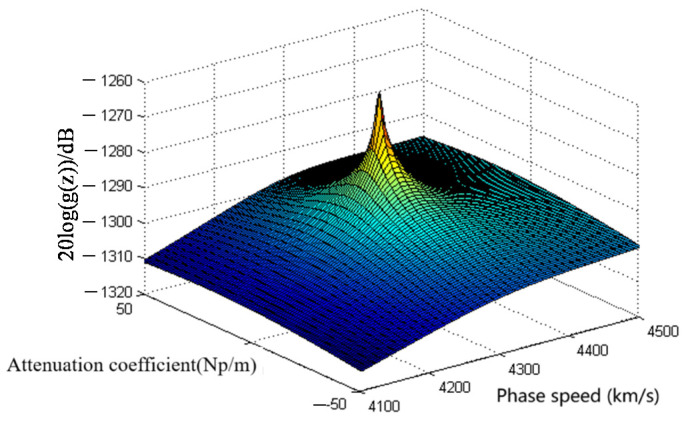
A local peak of *g*(*z*) formed by the zero of *f*(*z*).

Consider the function *g*(*z*), which is analytic over the whole complex plane, excluding some poles. The function has the characteristic that its absolute value is infinite at the poles and decreases rapidly when *z* is away from the poles. Therefore, as shown in Figure 2, if drawing a 3D image over the complex plane, a local peak appears around the poles in the image. Through searching the locations of local peaks, the complex domain of poles can be determined coarsely. Furthermore, the accurate value of poles can be obtained by the iteration method implemented among the determined coarse domains.

Firstly, the global domain search procedure is implemented. The location of local peaks of *g*(*z*) can be determined by a direct search method over the global discretized zone. First, it should select the zone to search, that is, determine the size of the complex plane to be discretized empirically, which should not be too large or too small, and the step used for discretizing the real and imaginary parts should be sufficiently small, in order to guarantee that all local peaks are included. When searched all over the selected zone globally, the coarse rectangle domains in the complex plane where local peaks locate are found by the direct search method totally.

**Figure 3:**
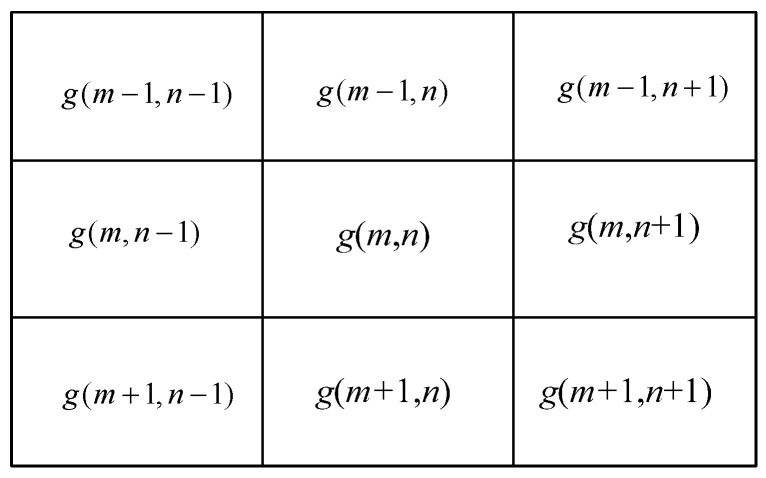
Window function w(i,j).

To find the local domains, it is needed to make sure there are local peaks inside. Assume the discretized values of *g*(*z*) are g(m,n), m,n=1,2,⋯; choose a 3 × 3 window function w(i,j):

(20) w(i,j)=g(m+i−2,n+j−2), i,j=1,2,3

where the terms of window function w(i,j) are shown in Figure 3.

If g(m,n) is the maximum of the 9 values inside the window, then g(m,n) might be the peak value of a local peak. When far away from the zeros, the value of the function changes slowly, while near the zeros, the value of the function changes dramatically. Using this characteristic, it is possible to determine whether it is a local peak or a small bump based on the speed of change of the function value. A metric Δ(*m*, *n*) is proposed, which can be used to measure the change speed of function value:

(21) $$\Delta \left(m,n\right)=\frac{g\left(m,n\right)-\left[\sum_{i,j}^{3}w\left(i,j\right)-g\left(m,n\right)\right]/8}{g\left(m,n\right)}$$

The reason for adopting this metric is that the value of *g*(*z*) varies rather rapidly close to the poles of *g*(*z*); however, if far away from the poles, the value will change smoothly. Based on this characteristic, Δ(*m*, *n*) can judge the slope of the function value changes. A parameter of ε > 0 is chosen empirically; when Δ(m,n)≥ε, the local peaks could be determined to exist, as seen in Figure 2. In the following, the kurtosis of ε is set to be 0.001. This value can be chosen empirically or experimentally. 

**Figure 4:**
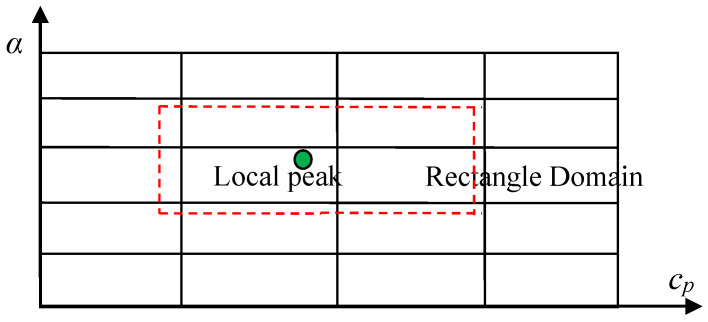
Rectangle domain containing the local peak.

When local domains containing local peaks are found, the local domain search procedure is implemented. The local peaks found are not very accurate, since they are just the approximate of the true results. In order to promote accuracy, the local peak is used as the centroid to establish a rectangle domain whose size is about several steps for discretizing the real and imaginary parts of the complex plane, as shown in Figure 4.

Next, to find the accurate solution of the dispersion equation, the Muller method is used to compute the value of the roots within the rectangle domain. The Muller method is suitable to solve the equation with one real or complex root inside a contour (i.e., the rectangle domain in Figure 4). The reason to use the Muller method is that, while keeping the least requirement for the dispersion function, its precision can be rather high. Thus, the discretization step has to be small enough to make every grid contain only one local peak. 

Finally, in order to guarantee the accuracy, a hierarchical scheme is established in the local domains; the iteration method is used to execute the scheme. When the coarse root is solved by Muller method, a refined rectangle domain around the root is set. Then the domain is discretized and the Muller method is used to refine the result again and again to make the error to be less than a given one, e.g., 10^−16^, which is also the stopping criteria of the iteration. In this way, a highly accurate result of the root can be obtained eventually. 

## 4. Software

The source code of this work is fully tested on various laminated structures under many boundary conditions, which are available on Github (see ‘Data Availability Statement’). In this section, the algorithm and the software are thoroughly described for usage. The source code is written in MATLAB programming language and tested on the R2022b version.

**Figure 5:**
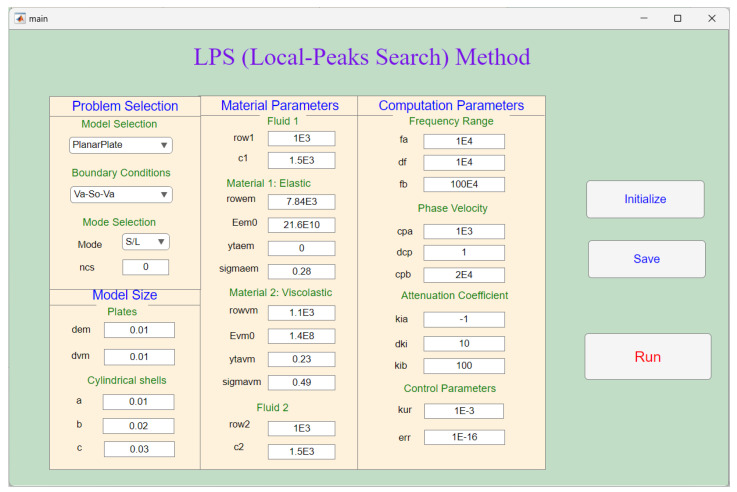
The main page for the LPS method based on MATLAB.

Figure 5 is the ‘main’ page of the software; it sets the parameters for the LPS method. For the ‘Problem Selection’ module, there are two different laminated structures to choose: one is the planar plate problem, and another is the cylindrical shell problem. Even though, in this paper, the planar plate problem is studied, the LPS method can also be applied for the cylindrical shell problems. The module of ‘Mode Selection’ is used only for cylindrical shell problems. For the ‘Model Size’ module, the thickness of the elastic and viscoelastic plates can be set; *a*, *b*, and *c* are the thickness of the elastic or viscoelastic cylindrical shells. For the ‘Material Parameters’ module, the parameters of fluid 1 and 2, elastic and viscoelastic materials are set. And the last module is ‘Computation Parameters’, in which the frequency range, the start, stop, and step parameters of the phase velocity, and the attenuation coefficient are set for discretization range of the complex plane. The ‘Control Parameter’ is set for determining the local peaks and the solution precision. 

**Figure 6:**
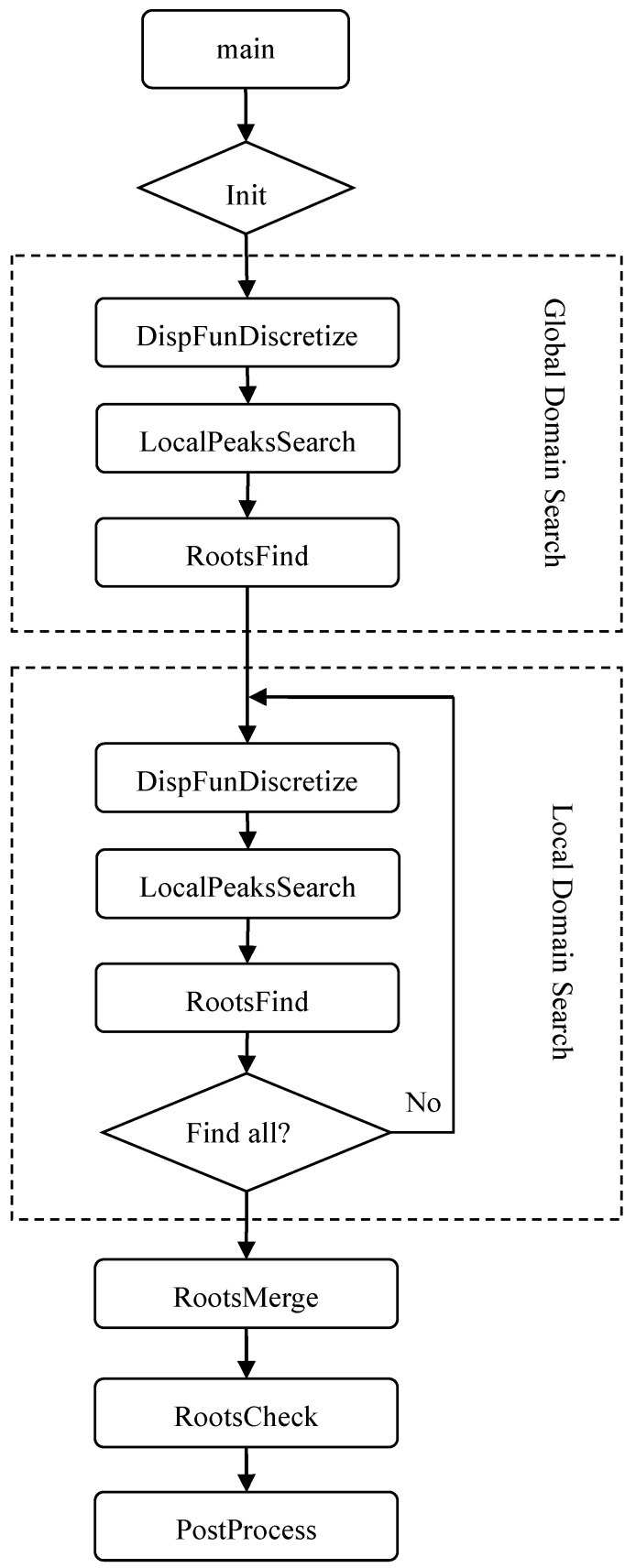
The algorithm of the LPS method program.

The architecture of the algorithm is shown in Figure 6; the algorithm is an implementation of the method depicted in Section 3. Through the main function, all parameters needed are initialized. Then, the global domain search procedure is executed, which first discretizes the selected domain over the complex plane with the phase velocity and the attenuation coefficient as the *x* and *y* axis, respectively. The result of discretization can be seen in Figure 2, which contains only one local peak. To find all of the local peaks, the direct search method is used, which is called ‘LocalPeaksSearch’ in the algorithm. After that, the roots-find method is applied here to obtain a relatively more accurate result for further usage. In this way, all of the local peaks in the region of interest are found.

Next, the local domain search method is executed; the procedures are the same as the global domain search. However, three procedures, i.e., the dispersion equation discretization, local peaks search, and the roots finding, are applied on the local domains around the local peaks. These three procedures are implemented to every single local peak. The error is controlled within the module ‘RootsFind’.

In order to obtain a highly accurate solution, a mixed scheme is designed. Along with the root-find method based on the Muller method, a domain refine method is proposed based on the hierarchical scheme in Section 3. That is, as we continue to discretize the local domain containing local peaks, its size will be reduced iteratively to less than the error. In this way, the roots can also be obtained accurately. The mixed scheme is then designed to combine the Muller root-find method and the domain refine method, which makes the algorithm much more robust.

After the local domain search is executed, if there are multiple roots, a scheme is established to remove all of the repeated ones. Then, a manual check module can be chosen to check whether the root is right or not. Of course, this manual procedure is not required and can be cancelled at the beginning. Finally, the post processing module can be used to plot the dispersion and attenuation curves.

## 5. Validation

For the computation and analysis of the Lamb waves’ dispersion curves, several different boundary conditions are selected to validate the LPS method. The material of the elastic plate is steel, its density is 7.84 × 10^3^ kg/m^3^, Young’s modulus is 3.0 × 10^7^ Pa, and the Poisson ratio is 0.28. 

**Figure 7:**
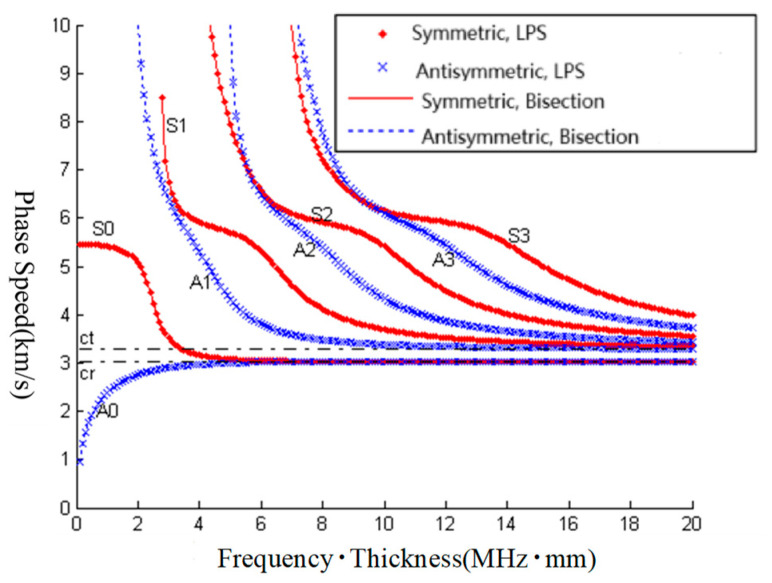
Comparison of the LPS method and bisection method.

As shown in Figure 7, for Lamb waves’ dispersion equations of free elastic plates, the bisection method can be used to compute the roots. The LPS method is compared with the bisection method in Figure 7; it can be seen that the results of the two methods are the same. Therefore, the accuracy of the LPS method is validated; it is highly accurate.

**Figure 8:**
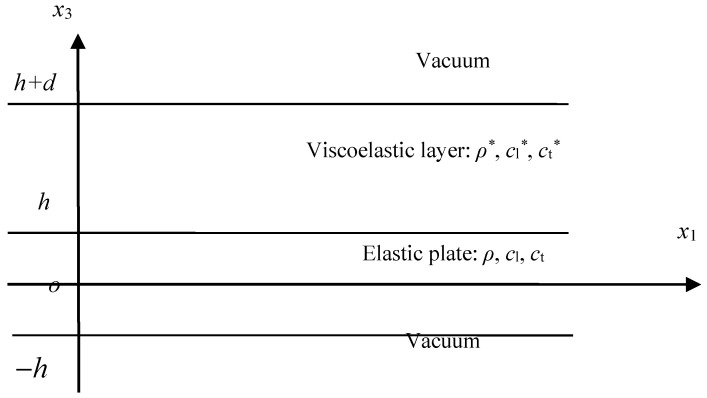
Lamb wave model of elastic plates coated with a viscoelastic layer in the vacuum.

It is absolutely a challenge to find a method to solve the complex dispersion equation of Lamb waves under diverse complicated boundary conditions. The bisection method failed in this problem, the LPS method, is applied to compute the dispersion and attenuation curves. To prove the potential of the LPS method to tackle such difficulties robustly, the dispersion equation of elastic plates coated with a viscoelastic layer in the vacuum is solved, as shown in Figure 8. The boundary conditions of the problem shown in Figure 8 can be written as

(22) $$\left\{ \begin{array}{l} {\sigma_{31}^{*}|}_{x_{3}=h+d}=0,{\sigma_{33}^{*}|}_{x_{3}=h+d}=0\\ {\sigma_{31}|}_{x_{3}=h}={\sigma_{31}^{*}|}_{x_{3}=h},{\sigma_{33}^{*}|}_{x_{3}=h}={\sigma_{33}|}_{x_{3}=h},{u_{1}|}_{x_{3}=h}={u_{1}^{*}|}_{x_{3}=h},{u_{3}|}_{x_{3}=h}={u_{3}^{*}|}_{x_{3}=h}\\ {\sigma_{31}|}_{x_{3}=-h}=0,{\sigma_{33}|}_{x_{3}=-h}=0 \end{array}\right.$$

By using the boundary conditions, the dispersion equation can be obtained:

(23) $$\left|D_{ij}\right|=0,i,j=1,\cdots ,8$$

in which the terms of the dispersion equation are given in Appendix A.

**Figure 9:**
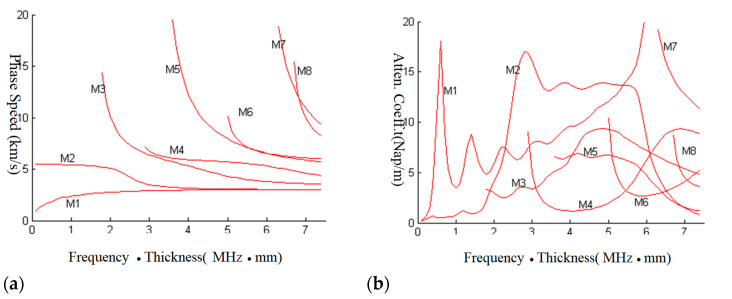
Dispersion and attenuation curves of Lamb waves from elastic plates with a viscoelastic layer in the vacuum. (**a**) Dispersion curve; (**b**) attenuation curve.

In the problem of Figure 8, the dispersion equation of the elastic plate coated with a viscoelastic damping layer in the vacuum is computed by the LPS method. The results of dispersion curves and attenuation curves are shown in Figure 9. In this problem, the symmetric and anti-symmetric modes of Lamb waves cannot be split; the first eight modes are computed. Furthermore, even though the modes cannot be split, the dispersion curves are somewhat like Figure 7, which means the viscoelastic layer is a load to the elastic plate and mainly influences the energy attenuation. The reason is that the impedance of the steel plate is much bigger than that of the viscoelastic layer.

## 6. Application

### 6.1. Highlight Model

**Figure 10:**
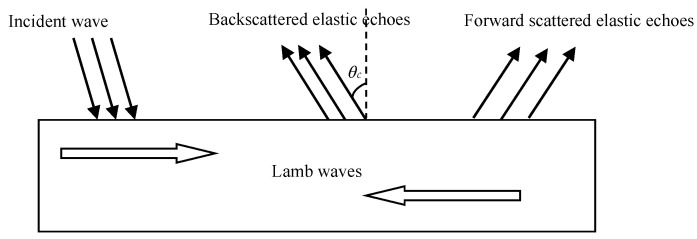
Echo structure of elastic plates in water.

For the active sonar, elastic scattering echoes are closely related to the propagation of acoustic waves in elastic bodies, so studying the propagation of acoustic waves in elastic plates and cylindrical shells can help in the understanding of the structure of target echoes, as shown in Figure 10. When a plane wave is incident on an elastic plate, a portion of the acoustic wave is reflected from the surface of the elastic plate to form a specular reflection wave. A part of the sound wave is scattered by the edges and corners of the elastic plate to form edge waves; another part of the sound wave penetrates into the elastic plate and excites the formation of Lamb waves. Due to the coupling between water and the elastic plate, the energy of the Lamb waves propagating in the plate leaks into the water, forming elastic scattered echoes, which is called the highlight model [19].

Considering elastic scattering echoes, the phase velocity of Lamb waves propagating in an elastic plate is denoted as $c_{p}$, while the phase velocity of acoustic waves in water is $c_{w}$. The boundary condition at the interface between water and the elastic plate requires the wavenumber of the acoustic wave to satisfy the continuity condition. Assuming that the angle between the elastic scattering wave and the normal of the boundary condition is

(24) $$\sin\theta_{c}=\frac{c_{w}}{c_{p}}$$

Along the direction of angle $\theta_{c}$, the received echo signal contains the elastic scattering echoes, while no elastic scattering wave is received in adjacent directions, resulting in a significant enhancement of the echo signal in the direction of $\theta_{c}$, known as the highlight points. $\theta_{c}$ is called the critical angle. Since Lamb waves are multi-modal, different modes have different critical angles for exiting into acoustic waves in water, leading to the observation of elastic highlights at multiple different angles.

### 6.2. The Experiment Deployment

**Figure 11:**
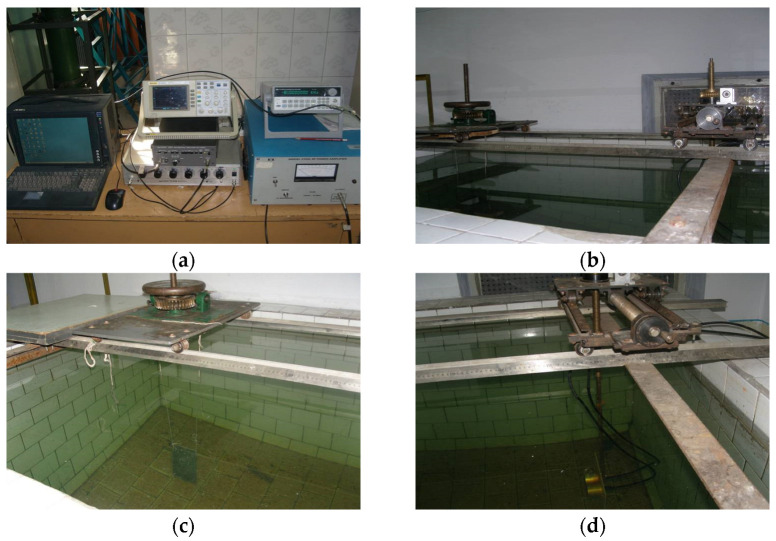
Diagram of connection and layout of experimental instruments and equipment. (**a**) The instruments for sound transmitting and receiving; (**b**) the mechanical equipment for deployment; (**c**) the deployment of the elastic plate; (**d**) the deployment of the monostatic sonar transducers (acoustic sensors).

The experiment was conducted in a tank, whose size is 2 m×1.5 m×1.5 m. Figure 11 shows the layout of the experimental instruments and equipment used in the experiment, as well as the arrangement of the transducer and the target. The transducer was installed using a transceiver configuration, and the target was suspended from a mechanic rotating device at the same depth as the transducer using a thin rope. The experiment is conducted by rotating the mechanical rotating device each step increased by one degree. The transmitting transducer emits a CW pulse, which is scattered by the elastic or laminated plate. The scattered waves are received by the receiving transducer. The mechanical device is rotated from 0 to 360 degrees, and experimental results are plotted in the time-angle pseudo-color image from which the highlights can be seen and determined.

**Figure 12:**
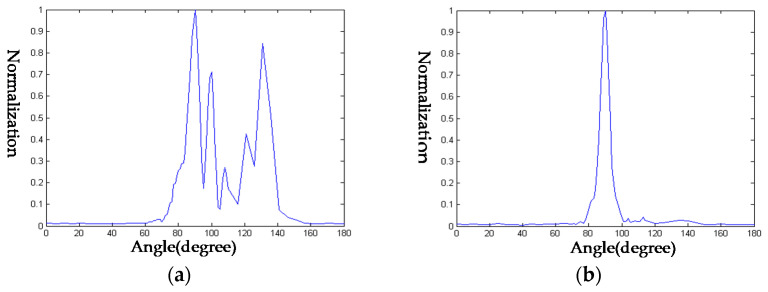

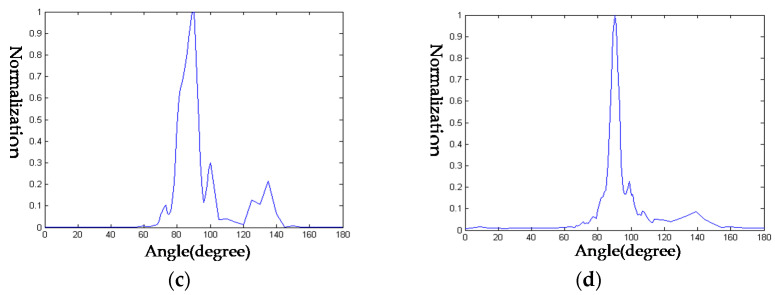
Directivity of A1 and A2. (**a**) Directivity of A1 at 800 kHz; (**b**) directivity of A1 at 1.1 MHz; (**c**) directivity of A2 at 800 kHz; (**d**) directivity of A2 at 1.1 MHz.

As shown in Figure 11d, a set of self-developed transducers in the laboratory, labeled A1 and A2, were used. One of these transducers was used as the transmitting transducer and the other as the receiving transducer. The receiving transducer was rotated to measure its directivity. The frequencies of the transmitted signals in the experiment were set at 800 kHz and 1.1 MHz, and the directivity obtained can be seen in Figure 12. At 800 kHz, the −3 dB beamwidth of transducer A1 was approximately $5.2^{\circ }$, and at 1.1 MHz, the −3 dB beamwidth was approximately $4.4^{\circ }$. For transducer A2, at 800 kHz the −3 dB beamwidth was approximately $8.2^{\circ }$, and at 1.1 MHz it was approximately $4.4^{\circ }$. From the directivity measurements of A1 and A2, it is evident that the directivity of A2 is superior to that of A1. Therefore, when conducting the target echo experiment, it is advisable to select A1 as the transmitter and A2 as the receiver.

### 6.3. Case for the Steel Plate

**Figure 13:**
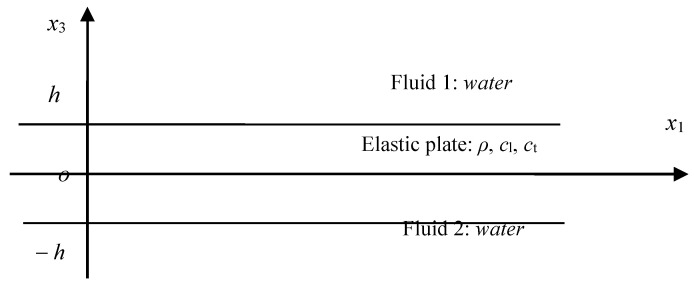
Leaky Lamb wave mode of elastic plates in water.

The first target is a steel plate, named as steel plate 1; its size was 150.02×131.82×1.07 m; its density was $\rho =7.84\times 10^{3}\mathrm{kg}/m^{3}$; Young’s modulus was $E=21.6\times 10^{10}\;N/m^{2}$; Poisson’s coefficient was σ=0.28. Figure 13 is the problem of the Lamb waves’ dispersion equation of the elastic plate in water; the boundary condition can be described as

(25) $$\left\{ \begin{array}{l} {\sigma_{31}|}_{x_{3}=h}=0,{\sigma_{33}|}_{x_{3}=h}=-{p_{1}|}_{x_{3}=h},{u_{3}|}_{x_{3}=h}={u_{3}^{1}|}_{x_{3}=h}\\ {\sigma_{31}|}_{x_{3}=-h}=0,{\sigma_{33}|}_{x_{3}=-h}={-p_{2}|}_{x_{3}=-h},{u_{3}|}_{x_{3}=-h}={u_{3}^{2}|}_{x_{3}=-h} \end{array}\right.$$

Under this boundary condition, the dispersion equation can be derived with the formal solutions. It can be found that, in this problem, just the same as the problem of elastic plates in vacuum, the vibration can be split into two different modes, i.e., the symmetric and anti-symmetric modes. The dispersion equation of the symmetric mode is

(26) $$\begin{array}{l} \left\{{\left(\beta^{2}-\xi^{2}\right)}^{2}\sin\left(\beta h\right)\cos\left(\alpha h\right)+4\xi^{2}\beta \alpha \sin\left(\alpha h\right)\cos\left(\beta h\right)\right\}\\ -i\frac{\rho_{0}\omega^{2}\alpha }{\mu \eta }\left\{\left(\beta^{2}+\xi^{2}\right)\sin(\alpha h)\sin\left(\beta h\right)\right\}=0 \end{array}$$

and the dispersion equation of the anti-symmetric mode is

(27) $$\begin{array}{l} \left\{{\left(\beta^{2}-\xi^{2}\right)}^{2}\sin\left(\alpha h\right)\cos\left(\beta h\right)+4\xi^{2}\alpha \beta \cos\left(\alpha h\right)\sin\left(\beta h\right)\right\}\\ +i\frac{\rho_{0}\omega^{2}\alpha }{\mu \eta }\left\{\left(\beta^{2}+\xi^{2}\right)\cos(\alpha h)\cos\left(\beta h\right)\right\}=0 \end{array}$$

**Figure 14:**
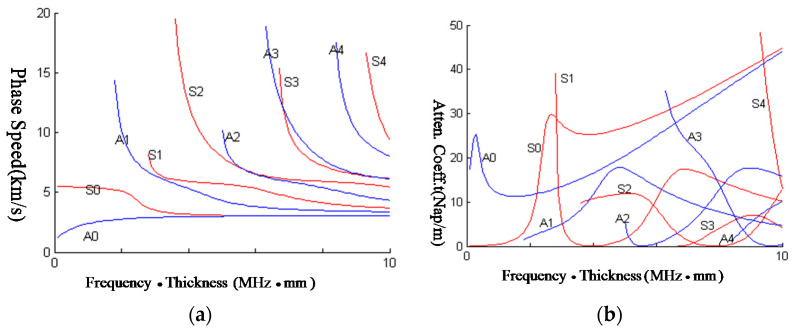
Dispersion and attenuation curves of Lamb waves from elastic plates in water. (**a**) Dispersion curve; (**b**) attenuation curve.

The dispersion equation of the steel plate in water is computed, as shown in Figure 14. In this problem, the water is served as the load to the plates, which will make the Lamb waves leaky; an attenuation coefficient is introduced to express the energy loss when Lamb waves propagate along the plate. However, the problem is symmetric; the Lamb waves can be split into symmetric and anti-symmetric modes. In this case, the dispersion function is complex, which results in complex roots of the solution. Using the LPS method to solve the problem, A0~A4 are the first five anti-symmetric modes and S0~S4 represent the first five symmetric modes.

**Figure 15:**
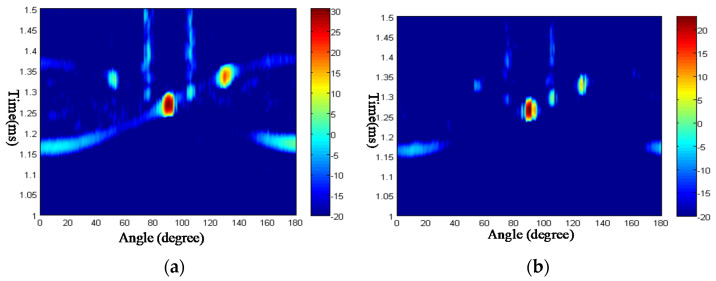
Time-angle pseudo-color picture of acoustic echoes from the steel plate. (**a**) Frequency 800 kHz; (**b**) frequency 1.1 MHz.

From Figure 15, it can be seen that there are several highlights in the time-angle figure, which is related to the Lamb waves by the critical angle. In Table 1, the numerical and experimental critical angles are compared, which demonstrates that the highlights are related to the elastic scattering wave components of the echoes. The elastic scattering wave components are coming from the leakage of Lamb waves, which is the mechanism of the acoustic echoes.

In Table 1, the phase speed of the Lamb wave is computed in the case shown in Figure 14. Using the formula of Equation (24), the critical angle is computed. From Figure 15, the critical angle can be determined by the highlights experimentally. Through comparison of the critical angle between numerical and experimental results, it can be seen the two results are close. This demonstrates the theory of the highlight model. 

**Figure 16:**
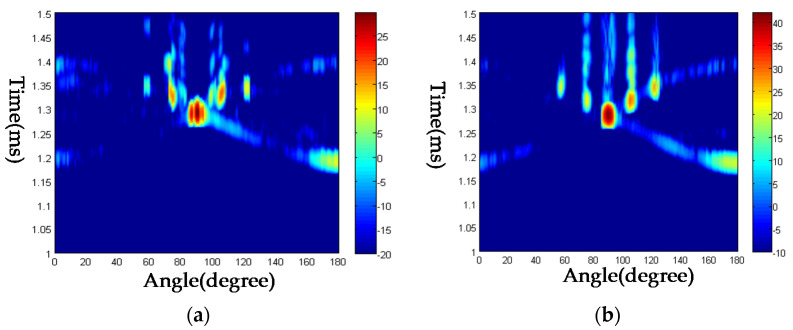
Time-angle pseudo-color picture of acoustic echoes from the steel plate 2. (**a**) Frequency 800 kHz; (**b**) frequency 1.1 MHz.

Another experiment is also conducted on a steel plate, named steel plate 2, whose size is 151.10 mm×133.20 mm×1.87 mm. After measuring the echoes by rotating the mechanic device from 0 to 180 degrees increased by one degree each step, the time-angle figure is obtained and drawn in Figure 16. The highlights can also be found in Figure 16a,b, the critical angles are determined, as shown in Table 2. The numerical results are also listed in Table 2, which are computed by the LPS method.

### 6.4. Case for the Steel Plate Coated with a Viscoelastic Layer

**Figure 17:**
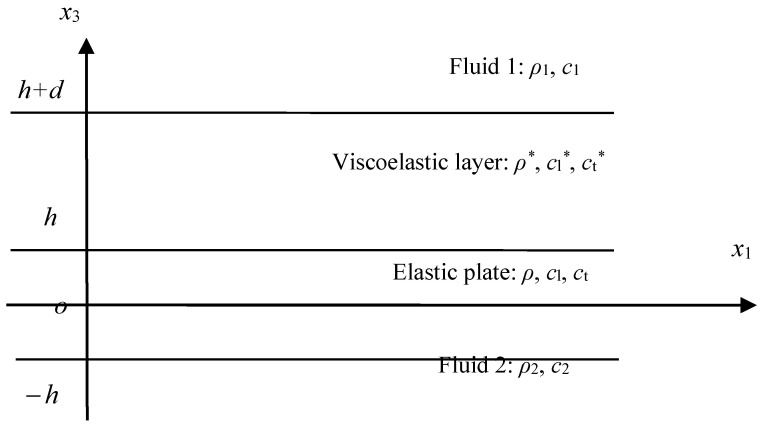
Lamb wave model for the elastic plate coated with viscoelastic layer in fluid.

In Figure 17, the material of the elastic plate is also steel, and it is coated with a viscoelastic layer, whose material is polyurethane. The size of the steel plate is 150.02 mm×131.82 mm×4.26 mm, and the thickness is 1.87 mm. For the viscoelastic material, its density is 1.1 × 10^3^ kg/m^3^, Young’s modulus is 21.6 × 10^10^ Pa, loss factor is 0.23, and Poisson’s ratio is 0.49; the thickness is 2.39 mm.

For the dispersion equation of the elastic coated with a viscoelastic damping layer under water, the boundary condition can be written as

(28) $$\left\{ \begin{array}{l} {\sigma_{31}^{*}|}_{x_{3}=h+d}=0,{\sigma_{33}^{*}|}_{x_{3}=h+d}={-p_{1}|}_{x_{3}=h+d},{u_{3}^{*}|}_{x_{3}=h+d}={u_{3}^{1}|}_{x_{3}=h+d}\\ {\sigma_{31}|}_{x_{3}=h}={\sigma_{31}^{*}|}_{x_{3}=h},{\sigma_{33}^{*}|}_{x_{3}=h}={\sigma_{33}|}_{x_{3}=h},{u_{1}|}_{x_{3}=h}={u_{1}^{*}|}_{x_{3}=h},{u_{3}|}_{x_{3}=h}={u_{3}^{*}|}_{x_{3}=h}\\ {\sigma_{31}|}_{x_{3}=-h}=0,{\sigma_{33}|}_{x_{3}=-h}=-{p_{2}|}_{x_{3}=-h},{u_{3}|}_{x_{3}=-h}={u_{3}^{2}|}_{x_{3}=-h} \end{array}\right.$$

By applying this boundary condition, the dispersion equation is derived:

(29) $$\left|D_{ij}\right|=0,i,j=1,\cdots ,10$$

in which the terms of the equation are given in Appendix B.

**Figure 18:**
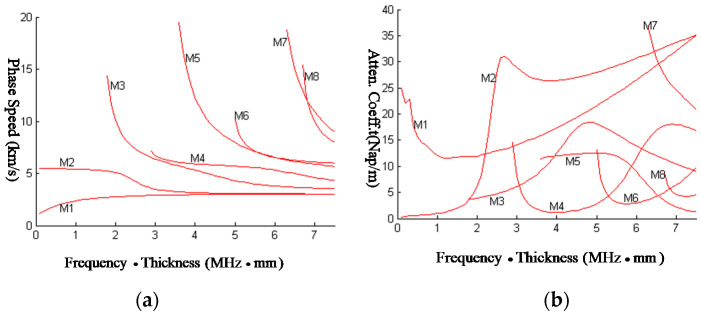
Dispersion and attenuation curves of Lamb waves from elastic plates coated with viscoelastic layer in water. (**a**) Dispersion curve; (**b**) attenuation curve.

This case is more complicated; however, the water is still a load to the elastic plates. In this problem, the coated plate is immersed in water, which can be further used to analyze the acoustic characteristics of elastic echoes scattered from the structure of plates in the water, as shown in Figure 18.

**Figure 19:**
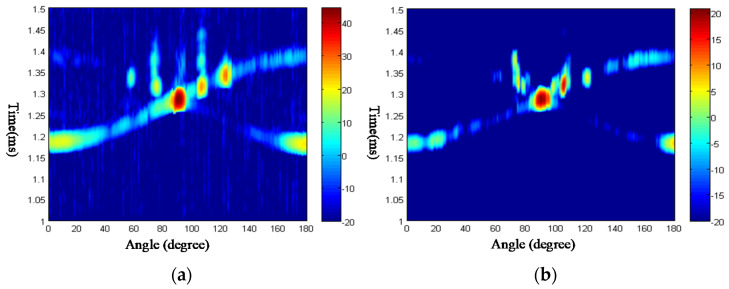
Degree-time pseudo picture of acoustic echoes from the steel plate coated with a viscoelastic layer. (**a**) Frequency 800 kHz; (**b**) frequency 1.1 MHz.

As shown in Figure 19, the data of acoustic scattering echoes is measured from the side of steel; the time-angle figure is obtained by experiment for different frequencies of 800 kHz and 1.1 MHz. From Figure 15, Figure 16 and Figure 19, it is possible to clearly observe the highlights of mirror reflection echoes, edge wave echoes, and elastic scattering echoes. Among them, the edge waves are reflected in the diagram as two bright lines, indicating that the scattering echoes produced by the edges can be received by the transducer at most angles. The two edge waves intersect at an angle position in the pseudo-color image, and their intersection is the mirror reflection echo. The highlights of elastic scattering echoes generated by Lamb waves can also be distinctly observed in the image, appearing on the bright lines produced by the edge waves.

From Figure 19, the critical angle can be determined by the highlights experimentally. The phase speed of the Lamb wave is computed in Figure 18, and then the critical angle is calculated afterward. In Table 3, through comparison of the critical angles between numerical and experimental results, it can further demonstrate the theory of the highlight model. However, some of the errors are large, caused by many factors that are analyzed in the next section.

### 6.5. Error Analysis

Table 1, Table 2 and Table 3 present the theoretical calculations and experimental measurements of the critical angles for the highlights of elastic scattering echoes for steel plate 1, steel plate 2, and the coated steel plate. It can be observed from the corresponding results in the tables that the theoretical calculations and experimental measurements are generally consistent, with a certain degree of error in the angles between the two. However, the errors are evidently within the acceptable range for engineering purposes. The errors between numerical and experimental critical angles can be several degrees. Some reasons for the errors are as follows:

1. The rotation device lacks precision, resulting in significant calibration errors on the turntable. When rotating one full circle, the actual rotation angle is approximately 9.73°, whereas theoretically it should be 10°. Additionally, the experiment only allows for rotation in increments of one large division (approximately 0.973° degrees, with 10 divisions in one full rotation), which lacks precision in angle adjustment;
2. Ensuring the accuracy of the corresponding angles in the experiment is challenging. For instance, the reflection point of the mirror should appear at angle 90°, but there are deviations observed in the experiment. This necessitates a certain degree of angle correction during the data processing, introducing errors in determining the critical angle value;
3. Due to the acoustic signal emitted by the transducer having a certain beamwidth, the elastic scattering highlights in the pseudo-color figure also exhibit some angular broadening, posing significant challenges in determining the critical angle size;
4. The power amplifier used in the experiment has some issues, resulting in signal distortion and inadequate power output, causing the transducer to generate weak acoustic waves and insufficient signal-to-noise ratio;
5. The material parameters used in the theoretical calculations are imprecise, leading to inaccurate calculation results that deviate from the measured experimental values.

## 7. Conclusions

This study on acoustic characteristics of sound propagated in or scattered from elastic plates and other laminated structures are related to Lamb waves. The key is to solve the dispersion equations of Lamb waves. Traditional root-finding methods are not able to tackle with the problem of solving dispersion equations of Lamb waves in the laminated structures totally, so a novel method named the LPS method is proposed to overcome their drawbacks while keeping their advantages.

The LPS method does not require computing the dispersion function’s derivatives or its orthogonality, etc.; the only requirement is that the absolute value of the dispersion function is continuous, which is the natural characteristic for the problem. Therefore, the LPS method might be considered as a universal root-finding method to solve problems of laminated structures whose dispersion equations can be expressed analytically or even any real and complex equation *f*(*z*) = 0 if the absolute value of *f*(*z*) is continuous. 

The work provides a fundamental tool for non-destructive testing, especially for underwater or industrial structural health monitoring. In this paper, the LPS software is applied to analyze the characteristics of the sound scattering from laminated structures of plates, i.e., the highlights formed by Lamb waves. The echoes are plotted in the time-angle figure to find and analyze the highlights and obtain the critic angle. Through computation of Lamb waves’ phase velocity, the critical angle is computed and compared with the experimental results, which can be used to predict the direction of highlights. The comparison reveals the mechanism of the elastic scattering wave components of the acoustic echoes, which are formed by the Lamb waves leaking into water. The correctness of the mechanism for the formation of elastic scattering echoes explained by the Lamb theory was validated by comparing the results of theoretical calculations at the critical angle with experimental measurements.

## Figures and Tables

**Table 1:** Highlights from steel plate 1.

Frequency	Mode Level	Phase Speed of Lamb Wave (m/s)	Numeric $\theta_{c}$	Experiment $\theta_{c}$	Error $\Delta \theta_{c}$
800 kHz	1	2278.95	41.2	39.9	1.3
	2	5430.81	16.0	15.1	0.9
1.1 MHz	1	2483.03	37.2	36.0	1.2
	2	5390.57	16.2	15.1	1.1

**Table 2:** Highlights from steel plate 2.

Frequency	Mode Level	Phase Speed of Lamb Wave (m/s)	Numeric $\theta_{c}$	Experiment $\theta_{c}$	Error $\Delta \theta_{c}$
800 kHz	1	2620.75	34.9	33.1	1.8
	2	5322.75	16.4	15.6	0.8
1.1 MHz	1	2774.42	32.7	32.1	0.6
	2	5026.49	17.4	15.6	1.8
	3	9552.86	9.0	8.8	0.2

**Table 3:** Highlights of the steel plate coated with a viscoelastic layer.

Frequency	Mode Level	Phase Speed of Lamb Wave (m/s)	Numeric $\theta_{c}$	Experiment $\theta_{c}$	Error $\Delta \theta_{c}$
800 kHz	1	2615.90	35.0	32.1	2.9
	2	5321.74	16.4	14.6	1.8
1.1 MHz	1	2770.38	32.8	29.2	3.6
	2	5023.04	17.4	14.6	2.8
	3	9545.80	9.0	8.8	0.2

## Data Availability

The source code of this work is available on Github under the MIT license: https://github.com/gjy2poincare/LocalPeaksSearchMethod, accessed on 24 September 2023.

## Appendix A

$$\begin{array}{l} D_{11}=D_{12}=D_{13}=D_{14}=0\\ D_{15}=-2i\xi \alpha^{*}\sin\left(\alpha^{*}d\right),D_{16}=2i\xi \alpha^{*}\cos\left(\alpha^{*}d\right)\\ D_{17}=\left(\beta^{*}{}^{2}-\xi^{2}\right)\cos\left(\beta^{*}d\right),D_{18}=\left(\beta^{*}{}^{2}-\xi^{2}\right)\sin\left(\beta^{*}d\right) \end{array}$$

$$\begin{array}{l} D_{21}=D_{22}=D_{23}=D_{24}=0\\ D_{25}=-\left(\beta^{*}{}^{2}-\xi^{2}\right)\cos\left(\alpha^{*}d\right),D_{26}=-\left(\beta^{*}{}^{2}-\xi^{2}\right)\sin\left(\alpha^{*}d\right)\\ D_{27}=-2i\xi \beta^{*}\sin\left(\beta^{*}d\right),D_{28}=2i\xi \beta^{*}\cos\left(\beta^{*}d\right) \end{array}$$

$$\begin{array}{l} D_{31}=-2i\xi \alpha \sin\left(\alpha h\right),D_{32}=2i\xi \alpha \cos\left(\alpha h\right),D_{33}=\left(\beta^{2}-\xi^{2}\right)\cos\left(\beta h\right)\\ D_{34}=\left(\beta^{2}-\xi^{2}\right)\sin\left(\beta h\right),D_{35}=0,D_{36}=-\frac{\mu^{*}}{\mu }2i\xi \alpha^{*}\\ D_{37}=-\frac{\mu^{*}}{\mu }\left(\beta^{*}{}^{2}-\xi^{2}\right),D_{38}=0 \end{array}$$

$$\begin{array}{l} D_{41}=-\left(\beta^{2}-\xi^{2}\right)\cos\left(\alpha h\right),D_{42}=-\left(\beta^{2}-\xi^{2}\right)\sin\left(\alpha h\right),D_{43}=-2i\xi \beta \sin\left(\beta h\right)\\ D_{44}=2i\xi \beta \cos\left(\beta h\right),D_{45}=\frac{\mu^{*}}{\mu }\left(\beta^{*}{}^{2}-\xi^{2}\right),D_{46}=D_{47}=0,D_{48}=-\frac{\mu^{*}}{\mu }2i\xi \beta^{*} \end{array}$$

$$\begin{array}{l} D_{51}=i\xi \cos(\alpha h),D_{52}=i\xi \sin(\alpha h),D_{53}=\beta \sin(\beta h)\\ D_{54}=-\beta \cos(\beta h),D_{55}=-i\xi ,D_{56}=D_{57}=0,D_{58}=\beta^{*} \end{array}$$

$$\begin{array}{l} D_{61}=-\alpha \sin(\alpha h),D_{62}=\alpha \cos(\alpha h),D_{63}=i\xi \cos(\beta h)\\ D_{64}=i\xi \sin(\beta h),D_{65}=0,D_{66}=-\alpha^{*},D_{67}=-i\xi ,D_{68}=0 \end{array}$$

$$\begin{array}{l} D_{71}=2i\xi \alpha \sin\left(\alpha h\right),D_{72}=2i\xi \alpha \cos\left(\alpha h\right),D_{73}=\left(\beta^{2}-\xi^{2}\right)\cos\left(\beta h\right)\\ D_{74}=-\left(\beta^{2}-\xi^{2}\right)\sin\left(\beta h\right),D_{75}=D_{76}=D_{77}=D_{78}=0 \end{array}$$

$$\begin{array}{l} D_{81}=-\left(\beta^{2}-\xi^{2}\right)\cos\left(\alpha h\right),D_{82}=\left(\beta^{2}-\xi^{2}\right)\sin\left(\alpha h\right),D_{83}=2i\xi \beta \sin\left(\beta h\right)\\ D_{84}=2i\xi \beta \cos\left(\beta h\right),D_{85}=D_{86}=D_{87}=D_{88}=0 \end{array}$$

## Appendix B

$$\begin{array}{l} D_{11}=D_{12}=D_{13}=D_{14}=0,D_{15}=-2i\xi \alpha^{*}\sin\left(\alpha^{*}d\right),D_{16}=2i\xi \alpha^{*}\cos\left(\alpha^{*}d\right)\\ D_{17}=\left(\beta^{*}{}^{2}-\xi^{2}\right)\cos\left(\beta^{*}d\right),D_{18}=\left(\beta^{*}{}^{2}-\xi^{2}\right)\sin\left(\beta^{*}d\right),D_{19}=0,D_{1,10}=0 \end{array}$$

$$\begin{array}{l} D_{21}=D_{22}=D_{23}=D_{24}=0,D_{25}=-\left(\beta^{*}{}^{2}-\xi^{2}\right)\cos\left(\alpha^{*}d\right)\\ D_{26}=-\left(\beta^{*}{}^{2}-\xi^{2}\right)\sin\left(\alpha^{*}d\right),D_{27}=-2i\xi \beta^{*}\sin\left(\beta^{*}d\right)\\ D_{28}=2i\xi \beta^{*}\cos\left(\beta^{*}d\right),D_{29}=\frac{\rho_{1}\omega^{2}}{\mu^{*}},D_{2,10}=0 \end{array}$$

$$\begin{array}{l} D_{31}=D_{32}=D_{33}=D_{34}=0,D_{35}=-\alpha^{*}\sin(\alpha^{*}d)\\ D_{36}=\alpha^{*}\cos(\alpha^{*}d),D_{37}=i\xi \cos(\beta^{*}d),D_{38}=i\xi \sin(\beta^{*}d),D_{39}=-i\eta_{1},D_{3,10}=0 \end{array}$$

$$\begin{array}{l} D_{41}=-2i\xi \alpha \sin\left(\alpha h\right),D_{42}=2i\xi \alpha \cos\left(\alpha h\right),D_{43}=\left(\beta^{2}-\xi^{2}\right)\cos\left(\beta h\right)\\ D_{44}=\left(\beta^{2}-\xi^{2}\right)\sin\left(\beta h\right),D_{45}=0,D_{46}=-\frac{\mu^{*}}{\mu }2i\xi \alpha^{*},D_{47}=-\frac{\mu^{*}}{\mu }\left(\beta^{*}{}^{2}-\xi^{2}\right)\\ D_{48}=0,D_{49}=0,D_{4,10}=0 \end{array}$$

$$\begin{array}{l} D_{51}=-\left(\beta^{2}-\xi^{2}\right)\cos\left(\alpha h\right),D_{52}=-\left(\beta^{2}-\xi^{2}\right)\sin\left(\alpha h\right),D_{53}=-2i\xi \beta \sin\left(\beta h\right)\\ D_{54}=2i\xi \beta \cos\left(\beta h\right),D_{55}=\frac{\mu^{*}}{\mu }\left(\beta^{*}{}^{2}-\xi^{2}\right),D_{56}=0,D_{57}=0\\ D_{58}=-\frac{\mu^{*}}{\mu }2i\xi \beta^{*},D_{59}=0,D_{5,10}=0 \end{array}$$

$$\begin{array}{l} D_{61}=i\xi \cos(\alpha h),D_{62}=i\xi \sin(\alpha h),D_{63}=\beta \sin(\beta h)\\ D_{64}=-\beta \cos(\beta h),D_{65}=-i\xi ,D_{66}=0,D_{67}=0,D_{68}=\beta^{*},D_{69}=0,D_{6,10}=0 \end{array}$$

$$\begin{array}{l} D_{71}=-\alpha \sin(\alpha h),D_{72}=\alpha \cos(\alpha h),D_{73}=i\xi \cos(\beta h),D_{74}=i\xi \sin(\beta h)\\ D_{75}=0,D_{76}=-\alpha^{*},D_{77}=-i\xi ,D_{78}=0,D_{79}=0,D_{7,10}=0 \end{array}$$

$$\begin{array}{l} D_{81}=2i\xi \alpha \sin\left(\alpha h\right),D_{82}=2i\xi \alpha \cos\left(\alpha h\right),D_{83}=\left(\beta^{2}-\xi^{2}\right)\cos\left(\beta h\right)\\ D_{84}=-\left(\beta^{2}-\xi^{2}\right)\sin\left(\beta h\right),D_{85}=D_{86}=D_{87}=D_{88}=D_{89}=D_{8,10}=0 \end{array}$$

$$\begin{array}{l} D_{91}=-\left(\beta^{2}-\xi^{2}\right)\cos\left(\alpha h\right),D_{92}=\left(\beta^{2}-\xi^{2}\right)\sin\left(\alpha h\right),D_{93}=2i\xi \beta \sin\left(\beta h\right)\\ D_{94}=2i\xi \beta \cos\left(\beta h\right),D_{95}=D_{96}=D_{97}=D_{98}=D_{99}=0,D_{9,10}=\frac{\rho_{2}\omega^{2}}{\mu } \end{array}$$

$$\begin{array}{c} D_{10,1}=\alpha \sin\left(\alpha h\right),\;D_{10,2}=\alpha \cos\left(\alpha h\right),\;D_{10,3}=i\xi \cos\left(\beta h\right)\\ D_{10,4}=-i\xi \beta \sin\left(\beta h\right),\;D_{10,5}=D_{10,6}=D_{10,7}=D_{10,8}=D_{10,9}=0,\;D_{10,10}=i\eta_{2} \end{array}$$
